# The role of arousal and motivation in emotional conflict resolution: Implications for spinal cord injury

**DOI:** 10.3389/fnhum.2022.927622

**Published:** 2022-10-05

**Authors:** Anna Pecchinenda, Adriana Patrizia Gonzalez Pizzio, Claudia Salera, Mariella Pazzaglia

**Affiliations:** ^1^Department of Psychology, Sapienza University of Rome, Rome, Italy; ^2^IRCCS Santa Lucia, Rome, Italy; ^3^Ph.D. Program in Behavioral Neuroscience, Sapienza University of Rome, Rome, Italy

**Keywords:** emotional conflict resolution, emotional interference, arousal, motivation, spinal cord injury, interoception, visceral

## Abstract

Under many conditions, emotional information is processed with priority and it may lead to cognitive conflict when it competes with task-relevant information. Accordingly, being able to ignore emotional information relies on cognitive control. The present perspective offers an integrative account of the mechanism that may underlie emotional conflict resolution in tasks involving response activation. We point to the contribution of emotional arousal and primed approach or avoidance motivation in accounting for emotional conflict resolution. We discuss the role of arousal in individuals with impairments in visceral pathways to the brain due to spinal cord lesions, as it may offer important insights into the “typical” mechanisms of emotional conflict control. We argue that a better understanding of emotional conflict control could be critical for adaptive and flexible behavior and has potential implications for the selection of appropriate therapeutic interventions.

## Introduction

Emotional information poses a challenge to our limited cognitive resources because it prioritizes attention and competes for processing resources, and because to achieve goal directed behavior, it may need to be ignored. Therefore, when emotional information is irrelevant to current goals, conflict arises and how it is resolved depends on different factors. Some factors pertain to the characteristics of the competing stimuli (e.g., low-level perceptual characteristics, perceptual salience, grouping, feature binding, etc.), but others directly tap onto cognitive control (see Pessoa, 2009; Pourtois et al., 2013). Albeit, implicit learning based on immediate (e.g., trial n-1 effects) or recent (e.g., contextual, statistical, and associative learning, etc.) history challenges this dichotomy between bottom-up and top-down factors. Interestingly, under certain conditions, emotional information reduces rather than enhances cognitive conflict.

The present perspective has three objectives: (i) to offer an integrative account of emotional conflict resolution; (ii) to define the contribution of emotional arousal and approach or avoidance motivation in accounting for conflict resolution; and (iii) to explore some of these mechanisms in patients with spinal cord lesion (SCI). We argue that a better understanding of the mechanisms underlying emotional conflict resolution could be critical for adaptive and flexible behavior and has potential implications for the selection of appropriate therapeutic interventions.

## Experimental task of emotional conflict

Research has typically used tasks that entail presenting task-relevant and task-irrelevant information either concurrently as two dimensions of the same stimulus (i.e., emotional variant of the Stroop task, Stroop, 1935), as two competing stimuli assembled as a compound (e.g., Word-Face Interference, Stenberg et al., 1998), or as two or more spatially separated stimuli (e.g., Flanker task, Eriksen and Eriksen, 1974).^1^ In the emotional variant of the Stroop task, a stimulus varies on two dimensions, one emotional and the other perceptual (e.g., emotional words written in different ink color), and participants respond based on the non-emotional, perceptual feature. In the emotional Word-face interference task, two stimuli (e.g., a word and a face) are presented superimposed onto the other (compound stimulus) one being the target and the other the distractor, whereas in the emotional Flanker task, target and distractor are presented next to each other.

Except for the emotional variant of the Stroop task and for some variants of the Flanker task, target and distractor can be affectively congruent (e.g., positive word and positive face) or incongruent (e.g., positive word and negative face). When a control condition is used, it entails presenting neutral in addition to the emotional distractors. Regardless of the specific task performed, emotional conflict is greater when the to-be attended and the to-be ignored stimuli (i.e., distractors) are affectively incongruent than when they are congruent or neutral. Importantly, emotional distractors yield a larger incongruence effect (i.e., interference) and greater performance impairment (i.e., slower responses and lower accuracy), compared to neutral distractors (e.g., Stenberg et al., 1998; Pecchinenda and Heil, 2007; Zhu et al., 2010; Strand et al., 2013; Pecchinenda et al., 2015; Ma et al., 2016; Petrucci and Pecchinenda, 2017; Viviani et al., 2021), independently of whether emotion is task relevant. Depending on the specific methodology used, this effect can stem from different underlying mechanisms, but response interference is always involved (Musch and Klauer, 2003). Importantly, in recent years, there has been evidence that emotional stimuli, rather than engendering greater interference, facilitate conflict resolution as there are faster and/or more accurate responses in the incongruent condition (for recent reviews, see Zinchenko et al., 2020). However, this finding is not homogenous probably due to differences in the methodologies used. This has focused the discussion on the factors affecting this effect and whether conflict resolution relies on phasic or tonic effects of emotion (Zinchenko et al., 2020). A recent review of available studies has concluded that evidence is so far inconclusive (Dignath et al., 2020). Moreover, that emotion facilitates conflict resolution has sometimes been attributed to the effect of arousal (Zeng et al., 2017; Landman and van Steenbergen, 2020). Taken together, this evidence points to the possible contribution of different underlying mechanisms to the effect of emotion on conflict resolution.

## Theoretical account of emotional conflict resolution

There are three major accounts for the mechanism responsible for emotion conflict resolution: *Dual-process theory, Dual-competition framework, Arousal-biased competition model:*

All three views received empirical support, suggesting that the *emotional conflict resolution* relies, in fact, on multiple factors. We combined this information to assess the role of emotional arousal and motivation in conflict resolution and whether they can operate independently or simultaneously.

### Dual-process theory: Reactive and proactive control

For the *dual-process theory* (Botvinick et al., 2001), there are two modes of cognitive control (or combinations of the two): *reactive control*, involved for instance in conflict adaptation, in which repetition of an incongruent trial increases cognitive control on the next trial, and *proactive control*, involved in anticipation of task demands. When applied to emotional conflict resolution (Botvinick, 2007), the account entails that as conflict yields negative affect, it increases reactive control and reduces emotional conflict. Therefore, especially in tasks relying on conflict adaptation and on trial-by-trial cognitive control requiring response inhibition, that negative stimuli facilitate emotional conflict resolution has been attributed to the emotional congruence between negative stimuli and the affect elicited by the conflict (Botvinick, 2007). However, in a recent review, Dignath et al. (2020) concluded that empirical support for the affective-signaling hypothesis is limited. Most importantly, this account would not explain that positive emotion facilitates emotional conflict resolution.

### Dual-competition framework

The *dual-competition framework* (Pessoa, 2009) proposes that the emotional and motivational salience of stimuli affects competition at both perceptual and executive levels (e.g., inhibition, shifting, and updating). Accordingly, emotional stimuli not only prioritize attention but also engage cognitive control, depending on whether they are relevant for the task at hand. When emotional stimuli are task-irrelevant, it is the level/intensity of threat or reward that affects whether they will engage cognitive control resources. This is achieved through connections between the amygdala, the anterior cingulate cortex, the striatum, and the dorsolateral prefrontal cortex. Importantly, the modulation of cognitive control would also affect visual processing. Therefore, emotion and motivation can impair or improve conflict resolution depending on interactive factors, albeit high intensity stimuli, including stimuli with high emotional arousal, divert cognitive resources from other mechanisms and impair performance. This account would not explain that high arousal, task irrelevant stimuli facilitate emotional conflict resolution.

### Arousal-biased competition model

The specific role of emotional arousal is the key aspect of the *arousal-biased competition model* (e.g., Knight and Mather, 2009), which accounts not only for emotion impairing but also for emotion enhancing cognitive control. Accordingly, depending on stimulus salience, emotional arousal amplifies the competition between stimuli to gain access to cognitive resources. Therefore, emotional arousal can enhance processing of goal-relevant or salient stimuli and impair processing of goal-irrelevant or less salient stimuli.

## Toward an integrated approach: Mechanisms of cognitive control and emotion regulation in emotional conflict resolution

Here, we argue for an integrated approach that considers the role of different factors on emotion conflict resolution, including emotional arousal and motivation. There is a long tradition of linking arousal to cognitive control. However, although cognitive control can be affected by arousal (see Porges, 2001), the direction of the effect and the underlying mechanisms are not well understood. For instance, increased arousal due to parasympathetic withdrawal and increased sympathetic activity has been linked to cognitive resources mobilization and better cognitive control. It has also been suggested that the arousal value of a stimulus, but not its valence, signals the urgency of processing and leads to motor preparation. Therefore, emotional arousal would facilitate processing of emotional stimuli, yielding faster responses (Robinson, 1998). It follows that if increased arousal due to parasympathetic withdrawal speeds up responses, then it may facilitate emotional conflict resolution by attenuating the slowing down due to response interference effect in tasks relying on response activation.

Importantly, the opposite relation between arousal and cognitive control has also been reported. Indeed, the neurovisceral integration model (Thayer et al., 2009) proposes that greater parasympathetic control of cardiac arousal is associated with better cognitive control as it reflects greater involvement of the prefrontal cortex, cingulate cortex, the medial prefrontal cortex, insula and of brain stem structures that regulate arousal (Thayer et al., 2012) and are involved in emotion regulation. It follows that higher resting values of heart rate variability (HRV) – an indicator of parasympathetic control over the heart – are linked to better cognitive control. Although this relationship has been predicted for tonic arousal, there is evidence that internal signals of moment by moment changes in arousal affect brain processing and cognitive control. For instance, Valt and Stürmer (2021) showed that enhanced phasic physiological arousal is evoked not only by incorrect responses but also by correct responses and this phasic arousal guides optimal performance on a trial by trail basis during cognitive tasks. Importantly, the effects of these internal signals seem to be independent of valence (see also the reinforcement-learning model of Holroyd and Coles, 2002 for the role of dopaminergic signals to the anterior cingulate cortex depending on performance).

We propose to re-conceptualize the effect of emotion on conflict resolution in terms of mechanisms of implicit cognitive control and emotion regulation linked to arousal. Not only cognitive control is enhanced by efficient emotion regulation (Teper et al., 2013) but emotional conflict resolution may call upon the same processes and mechanisms. These interactive effects would not be surprising considering that emotion regulation (e.g., attentional deployment, distraction, and selection all rely on attention, Gross and Thompson, 2007) and cognitive control rely on shared neural circuits (e.g., Phan and Sripada, 2013). In this case, emotional arousal alone would facilitate conflict resolution by enhancing cognitive monitoring and attentional resources allocation (see also Ahumada-Méndez et al., 2022). Depending on whether the to-be attended and the to-be ignored emotional information belong to the same (e.g., allowing for perceptual grouping) or different stimuli, emotion regulation strategy may rely on selectively attending to one aspect of the stimulus over another, shifting attention away from one stimulus toward another, inhibiting the activated responses, or combinations of different strategies.^2^ In contrast, when the to-be ignored and to-be attended emotional stimuli co-occur, emotional conflict resolution may be achieved by means of response-focused regulatory strategies (i.e., response inhibition). However, the success of response inhibition may depend not only on the elicited emotional intensity/arousal, but also on the activated motivational tendency, such that it may be more difficult to inhibit an activated approach response compared to when avoidance is primed, especially when emotional arousal is high, and emotion is task-relevant.

In the next section we discuss the role of internal signals and phasic arousal generated during emotional conflict in patients with SCI.

## Emotional and cognitive consequences of autonomic dysregulation after spinal cord injury

That emotion regulation may rely on tonic or on moment-by-moment physiological arousal *via* interoceptive pathways (Thayer and Lane, 2000; Porges, 2001), and that interoceptive feedback may also intrinsically influence emotion regulation processes, by acting at the autonomic level (Damasio and Carvalho, 2013). Of interest here is whether emotion regulation may be attenuated when the autonomic flow of information between the brain and body is permanently disrupted. As the primary relay center of neural transmission, a SCI interrupts the communication of the afferent and efferent systems between the brain and the rest of the body (Leemhuis et al., 2019, 2022), *via* the spinal projections (Bican et al., 2013; Rupp, 2020). The axotomy after SCI has thus implications for the motor, sensory, autonomic physiology of the cortical and corticospinal network (Nagendran et al., 2017; Michael et al., 2019; Benedetti et al., 2022). Accordingly, patients with SCI represent an ideal model for studying the role of physiological arousal in resolving emotional conflict, given their distinct pathophysiological autonomic profile.

More specifically, higher SCI [at or above the 6th thoracic segment (≥ T6)] due to disruption of sympathetic nervous system pathways (Krassioukov, 2009; Craig et al., 2018), result in an imbalance of sympathetic and parasympathetic activity (Teasell et al., 2000). The loss of supraspinal sympathetic nervous system input following high-level SCI leads to systemic hypotension and episodes of uncontrolled hypertension (Krassioukov, 2009) with an impaired hemodynamic balance which is negatively affected by postural stress (Teasell et al., 2000). Individuals with SCI ≥ T6 may be at higher risk than those with lower lesions to develop autonomic alterations, including heart rate, blood pressure, skin temperature, gut motility, respiration, and piloerection, that are critical for neurophysiological regulation of emotional responses (Nicotra et al., 2006; Migliorini et al., 2009; Mather and Thayer, 2018).

### The loss of physiological arousal and its effects on emotion and cognition after spinal cord lesion

Several available SCI evidence suggests that emotional experience is markedly reduced in comparison to pre-injury SCI (Hohmann, 1966; Lowe and Carroll, 1985; Chwalisz et al., 1988). It has been reported that individuals with complete cervical SCI have difficulties in recognizing fear- and anger-inducing scenes (Pistoia et al., 2015), and report less anxiety and less intense emotions compared to healthy individuals (Montoya and Schandry, 1994). The intensity of emotional responses is associated with decreased cardiac awareness in patients with complete SCI between C5 and T4, which highlights the importance of interoceptive feedback for emotional experience, especially in complete higher SCI (Montoya and Schandry, 1994). When they are exposed to arousing slides of sexual content, individuals with higher lesions express less intense feelings of arousal than individuals with lower lesions (Jasnos and Hakmiller, 1975). Also, expressed arousal in situation of high and low emotional relevance is similar in individuals with cervical lesions but differs in those with thoracic and lumbar lesions (Jasnos and Hakmiller, 1975). Autonomic arousal feedback may also play some role in the cognitive impairment that occurs in 60% of younger and older individuals with SCI, with deficits documented in attention, concentration, abstract reasoning, and processing speed (Davidoff et al., 1985, 1990; Roth et al., 1989; Dowler et al., 1995, 1997; Jegede et al., 2010; Chiaravalloti et al., 2020). Existing studies suggest that in the absence of brain damage, physiological variables are among the factors that more impact at least one cognitive domain after SCI (Sachdeva et al., 2018; Wecht et al., 2018; Nightingale et al., 2020). Lowered HRV and systolic blood pressure are two of the most important factors explaining the link between autonomic cardiovascular dysfunctions and cognitive response in SCI (Wecht et al., 2018; Sachdeva et al., 2018; Nightingale et al., 2020). People with high-level SCI (≥ T6) perform worse cognitively because of unstable cardiovascular activity (Wecht et al., 2018; Nightingale et al., 2020) and poor emotional and stress control (Varas-Díaz et al., 2017) during cognitive performance (Wecht et al., 2018). These changes are primarily seen when evaluating information processing speed, sustained attention, and executive function (Jegede et al., 2010; Wecht et al., 2018; Nightingale et al., 2020). More specifically, the unstable fluctuations in systemic blood pressure are relevant for the impairment of attention and processing speed in people with SCI ≥ T6 (Nightingale et al., 2020). Additionally, the hypotensive SCI group is more impaired in memory and attention compared to the normotensive group (Jegede et al., 2010). Therefore, chronic adrenergic denervation among SCI ≥ T6 can have significantly reduced the effect of emotional valence and arousal on long-term memory (Cahill et al., 1994; Reist et al., 2001; van Stegeren et al., 2002). Indeed, online tracking of transient episodes of hypotension and hypertension during cognitive performance found that a patient with non-traumatic cervical SCI showed a rapid drop in blood pressure as well as decreased cardiovascular parameters, which were associated with a decreased response rate and an increase in the number of incorrect answers on an arithmetic test (Kjaerup et al., 2021). Together, these investigations point to an autonomic mechanism that is unable to support patients with severe SCI injuries during simple or more challenging cognitive tasks. The implication being that internal signals based on moment-to-moment arousal changes are compromised in patients with SCI caused by high lesions but not in patients with SCI caused by low lesions. Concerning the role of avoidance and approach motivation in contributing to emotional conflict resolution, this also may be compromised in individuals with SCI, who find it difficult to recognize primary physiological needs (Lucci and Pazzaglia, 2015), exhibit low interoceptive sensitivity and feel a greater sense of detachment from their internal sensations (Lenggenhager et al., 2012; Salvioli et al., 2012). In the next section, we propose that the two types of SCI lesions (high vs. low) may differently affect emotional conflict resolution due to the involvement of physiological arousal alterations.

### A pathophysiological autonomic spinal cord lesion profile for understanding the role of arousal and motivation in resolving emotional conflict

It has been assumed that normal psychological coping responses to tragedy and loss, adequately explain the cognitive and emotional alterations observed after SCI (Grassman et al., 2012, Klyce et al., 2015; Jenkins and Cosco, 2021).

However, there has not been any debate in the scientific literature regarding how physiological changes following the lesion may, at least partially, influence emotional/cognitive conflict in SCI (Hohmann, 1966; Jasnos and Hakmiller, 1975; Montoya and Schandry, 1994; Cobos et al., 2004).

In this perspective, we have highlighted that, changes in emotional and cognitive abilities differ with the degree and level of SCI lesion; it is generally greater as the injury progresses up the spinal cord, and it is more pronounced in patients with complete injuries. Although autonomic alterations and body dysregulation widely contribute to cognitive dysfunction after SCI (Hou and Rabchevsky, 2014; Sachdeva et al., 2018), there are relatively few studies that have differentiated the specific pathophysiological profiles of patients with SCI (Pazzaglia et al., 2018).

We hypothesized that, beyond the negative psychological reaction to injury and individual resilience, there could be a trend toward specific neurocognitive and emotional behavioral variations among patients with SCI and that these might be connected to the anatomic level of cord injury. However, no studies have directly investigated the effects of autonomic reactivity induced by the severity of lesion on cognitive and emotional functions. We briefly documented a number of empirical evidences highlighting the role played by the level of the SCI in cognitive and emotional processing. In paragraph 5.1 we discussed cognitive changes of patients SCI ≥ T6 on tasks indirectly relying on processing speed (e.g., Dowler et al., 1995), response inhibition and sustained attention when feedback is provided (Davidoff et al., 1985). These findings suggest that phasic physiological arousal evoked on a trial by trial basis during cognitive tasks – e.g., by response feedback (Valt and Stürmer, 2021) – cannot guide optimal performance. We pose that arousal and motivation play an important role in resolving emotional conflict in SCI. However, this has not yet been explicitly tested. We suggest that, well defined cognitive tasks on the effect of arousal and motivation on emotion conflict resolution should be used in patients with SCI. Future studies investigating to what extent sympathetic dysregulation in individuals with SCI performing different tasks (Flanker, Stroop, and Word-Face Interference task) can contribute to clarify the role of arousal and motivation in resolving emotional conflict and, to what extent it is affected by autonomic dysregulation rather than by sensory and motor deafferentation. To contribute substantially to disentangling the role of arousal and motivation in emotional conflict resolution, the studies should include: (1) accurate comparison of homogeneous samples of patients with complete, chronic, and SCI ≥ T6 lesions compared to lower lesions; and (2) trial by trial analyses (i.e., n-1 trial effects) synchronized with HRV of patients should be tested. HRV is currently used only occasionally in experiments that are designed to assess cognitive and emotional functioning of patients with SCI but no research has yet verified its validity to assess the role of arousal states. We suggest HRV monitoring, as it reflects the state of autonomic nerve system, and may be considered a promising early biomarker of cognitive and emotional impairment in populations with SCI. If the altered cognitive-emotional processes of individuals with SCI stem from impaired implicit biases due to physiological arousal and activation of motivational tendencies, this poses a serious challenge not merely in the rehabilitation phase (Leemhuis et al., 2021). Disentangling the different factors affecting emotion and cognitive functions in SCI, including the physiological changes brought on by the lesion, will make obvious differences to treatment (Klyce et al., 2015), re-employment, and reintegration into society (Scivoletto et al., 2019).

## Conclusion

The present perspective offers an integrative account of the mechanisms that may underlie the resolution of emotional conflicts in tasks involving response activation, highlighting the contribution of emotional arousal and primed approach/avoidance. Direct evidence on the contribution of emotional arousal to cognitive control may come from SCI studies, providing the simplest conceivable test for the hypothesis that arousal biases emotional and cognitive control. In fact, if arousal, together with approach and avoidance tendencies, affect emotional conflict resolution, then an effect of SCI lesion level might be predicted.

## Data availability statement

The original contributions presented in the study are included in the article/supplementary material, further inquiries can be directed to the corresponding authors.

## Ethics statement

Ethical review and approval was not required for the study on human participants in accordance with the local legislation and institutional requirements. Written informed consent for participation was not required for this study in accordance with the national legislation and the institutional requirements.

## Author contributions

AP and MP: conceptualization and supervision. AP, MP, APGP, and CS: writing – original draft preparation and review, and editing. All authors have read and agreed to the published version of the manuscript.

## Footnotes

1 The description is for the visual modality but the logic can be applied to target and distractors presented in auditory and visual modalities.

2 Under some conditions, when the Flanker task entails perceptual load, emotional conflict resolution may result from high perceptual load (see Lavie, 2005).
